# Journey Through the World of Hair

**DOI:** 10.4103/0974-7753.51918

**Published:** 2009

**Authors:** Sundaram Murugusundram

**Affiliations:** Consultant Dermatologist/Secretary, The Hair Research Society of India, Chennai, India

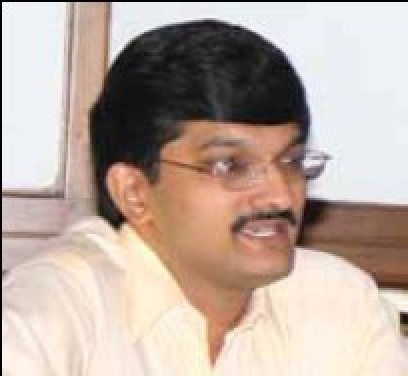



Exactly five years ago a little flower in the garden of wisdom blossomed as the Hair Research Society of India. Early in 2004, when I registered to participate in the "World of Hair"- fourth intercontinental meeting of hair research societies in Berlin, I was asked to contact the hair research society of my country for a letter of recommendation. Having discovered that such an organization did not exist in my country, I sought the help of my teacher Prof. Patrick Yesudian who had been a "lead kindly light" in all my endeavors. Giving no room for second thought said he, "why not we?" The seed sown by him was nurtured into a sapling by Prof. S. Shivakumar and enthusiastic colleagues with the thirst for knowledge.

On 13 June 2004, from the land of diverse culture, history and mystery the Hair Research Society of India was started with the aims of gaining and disseminating the knowledge of hair with the transformation of the same into safe and ethical practices in order to abolish quackery and promote science. It was decided to conduct quarterly meetings with guest lectures by inter-disciplinary specialists, clinical case discussions and to publish a newsletter which would carry the proceedings of the meeting.

It was Prof. Rudolph Happle, the king of limericks who *"when confronted with matters genetic; inclined to be somewhat frenetic; thought to be archaic; invokes a mosaic; with Blashco becomes energetic"*, wanted the Hair Research Society of India to be a "Growing Hair Society" and not simply a hair-growing society.

Our Indian hair newsletter used to be like a sumptuous dinner with a starter of a short note on the current trichological delicacy by our president Prof. Patrick Yesudian, a main course of a highly informative guest lecture and a cool dessert of challenging clinical hair disorders. Prof Urlike Blume-Peytavi, the past president of the European Hair Research Society (EHRS) appreciated our intention of stimulating our colleagues with the knowledge of trichology and encouraged photo documentation of clinical hair disorders in the Indian scenario.

Having conducted 20 quarterly meetings and published 20 newsletters, the Hair Research Society of India has reached yet another milestone in the journey of academic pursuit with the launch of the *International Journal of Trichology*, the world′s first dedicated peer-reviewed journal for hair. The journal is fortunate to have a genius like Prof. Desmond J Tobin to be the executive editor who had designed the creative cover page of the journal so meticulously. The editorial board glitters with the luminaries in trichology across the globe.

Inspired by the EHRS, the Mecca of hair science, the Hair Research Society of India has come a small way in this half a decade. At this moment I thank the presidents, members, researchers, colleagues and patients from whom we learn. The joy of learning continues as saint *Thiruvalluvar* says in *Thirukural*, a repertory of universal thoughts and truths, *"As deep you dig the sand spring flows; As deep you learn the knowledge grows."*

